# Developments in the Application of Nanomaterials for Water Treatment and Their Impact on the Environment

**DOI:** 10.3390/nano10091764

**Published:** 2020-09-07

**Authors:** Haleema Saleem, Syed Javaid Zaidi

**Affiliations:** Center for Advanced Materials (CAM), Qatar University, P.O. Box 2713 Doha, Qatar; haleema.saleem@qu.edu.qa

**Keywords:** nanomaterials, water pollution, adsorbents, membranes, toxicity, environment

## Abstract

Nanotechnology is an uppermost priority area of research in several nations presently because of its enormous capability and financial impact. One of the most promising environmental utilizations of nanotechnology has been in water treatment and remediation where various nanomaterials can purify water by means of several mechanisms inclusive of the adsorption of dyes, heavy metals, and other pollutants, inactivation and removal of pathogens, and conversion of harmful materials into less harmful compounds. To achieve this, nanomaterials have been generated in several shapes, integrated to form different composites and functionalized with active components. Additionally, the nanomaterials have been added to membranes that can assist to improve the water treatment efficiency. In this paper, we have discussed the advantages of nanomaterials in applications such as adsorbents (removal of dyes, heavy metals, pharmaceuticals, and organic contaminants from water), membrane materials, catalytic utilization, and microbial decontamination. We discuss the different carbon-based nanomaterials (carbon nanotubes, graphene, graphene oxide, fullerenes, etc.), and metal and metal-oxide based nanomaterials (zinc-oxide, titanium dioxide, nano zerovalent iron, etc.) for the water treatment application. It can be noted that the nanomaterials have the ability for improving the environmental remediation system. The examination of different studies confirmed that out of the various nanomaterials, graphene and its derivatives (e.g., reduced graphene oxide, graphene oxide, graphene-based metals, and graphene-based metal oxides) with huge surface area and increased purity, outstanding environmental compatibility and selectivity, display high absorption capability as they trap electrons, avoiding their recombination. Additionally, we discussed the negative impacts of nanomaterials such as membrane damage and cell damage to the living beings in the aqueous environment. Acknowledgment of the possible benefits and inadvertent hazards of nanomaterials to the environment is important for pursuing their future advancement.

## 1. Introduction

Nanotechnology characterizes a groundbreaking pathway for scientific development that concerns the managing of material at the nanoscale level (a nanometer is 10^−9^ of a meter) [1]. Nanotechnology precisely means any technology on the nanometer scale, which has several applications in the real world. This technology factually includes the manufacture as well as the application of biological, physical, and chemical systems at scales varying from distinct molecules or atoms to submicron dimensions, and the incorporation of these resultant nanomaterials (NMs) into bigger systems. Nanomaterials comprise of one or more structural dimensions at the nanoscale and have given rise to extreme research interest because of its utilization possibility in various areas of science as well as technology. The distinguishing structures of nanomaterials should fall between bulk materials and single atoms. Accordingly, the nanomaterials mostly demonstrate exclusive and remarkably enhanced properties, however occasionally unpredictable biological, chemical, and physical characteristics contrasting with their bulk materials [2].

In spite of such developments in nanomaterial technology, details regarding the possible effects of NMs on the environment as well as human health are until now inadequate [3]. Due to the fact that the nanomaterials might not be noticeable after its release into the surroundings, these materials can lead to different kinds of environmental issues if the remediation strategy is unsecured. Consequently, extra study is essential to scientifically describe the structure–function correlation of nanomaterials with regard to its fundamental chemistry (for example, toxicity as well as functionality). Furthermore, complete risk evaluations need to be carried out on nanomaterials, which present a real exposure danger in the course of production or utilization. Henceforth, green nanoscience has been recommended in order to diminish the possible human health and environmental threats from the manufacture and utilization of NMs and to develop the replacement of prevailing items with advanced nanomaterials, which are more environmentally friendly [4].

Recently, the developing nanotechnology contributes a prospective offer for purifying water with an economical, superior operating efficiency in eliminating contaminants and reusable capability [5]. In recent times, as the world faces scarcity for potable water, scientists have proved that nanomaterials will be a superior option for treating wastewater, due to the fact that it has certain exclusive characteristics such as a huge surface area, nano size, extremely reactive, good mechanical stability, powerful solution mobility [6], dispersibility, hydrophilicity, and hydrophobicity [7,8,9]. Certain heavy metals (such as Pb, Mo, etc.), numerous harmful microbes, inorganic and organic contaminants are reported to be effectively eliminated by utilizing diverse nanomaterials [10,11,12]. Lately, more advancements have occurred in nanomaterials like nanomembranes, nanophotocatalysts, and nanosorbents, which could be efficiently utilized for the treatment of polluted water. In brief, the proper analysis of nanomaterial application in water treatment is considered to measure the positive outlooks.

This review paper focuses on the application of different nanomaterials in the aqueous environment for several environmental remediation applications. In this paper, we will analyze the benefits and drawbacks of frequently utilized carbon-based nanofillers (carbon nanotubes and graphene) along with metal as well as metal oxides based nanofillers (based on zinc oxide, titanium dioxide, silver, and nano zerovalent iron) exposed to the environment. We believe that this paper would contribute new understandings and knowledge for the scientific community in this field. This will also contribute to the exploration of more advanced nanomaterials for improving water treatment efficiency. We have found some papers providing an overview of nanomaterials used for water treatment [13,14,15,16,17]. However, to the best of our knowledge, there are not many works discussing the latest developments in nanotechnology for environmental remediation application in water, advantages of nanomaterials in applications such as adsorbents (removal of dyes, heavy metals, pharmaceuticals, and organic contaminants from water), membrane materials, catalytic utilization, and microbial decontamination, along with its related disadvantages, mainly due to the toxicity of nanomaterials and their ability to modulate toxic effects of other substances. We have reviewed the dangerous effects of the nanomaterials on aquatic plants and marine organisms. Furthermore, different factors influencing the efficiency of the nanomaterials are also analyzed. We mainly discuss the negative impact of nanomaterials such as CNTs, silver nanoparticles, and graphene-based nanomaterials in the aqueous environment.

## 2. Different Types and Properties of Nanomaterials

**Figure 1:**
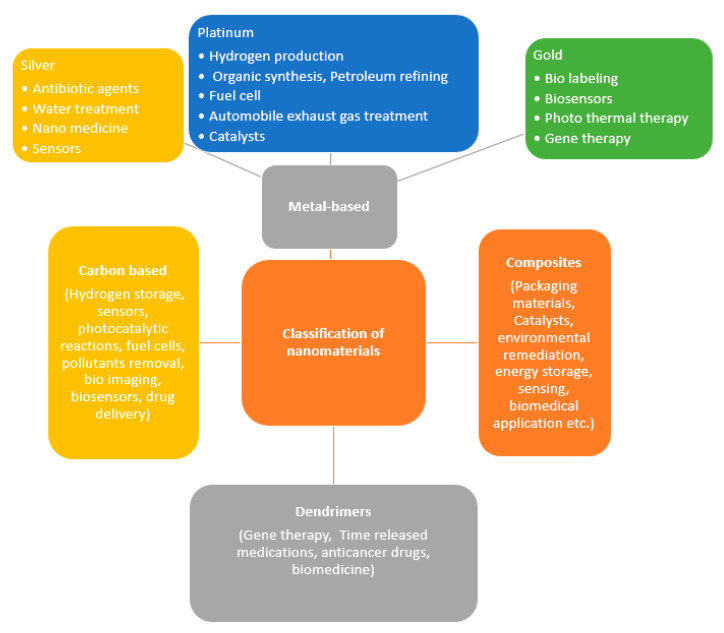
Different classification as well as the extensive range of applications of some of the nanomaterials.

Structures having dimensions of 1–100 nm are generally considered as the nanomaterials [18]. Due to its larger surface-area-to-volume ratio and the possible existence of quantum effects, the nanomaterials perform relatively different from their bulk counterparts [19]. The nanostructure materials can be classified as zero-dimensional (0-D), one-dimensional (1-D), two-dimensional (2-D), and three-dimensional (3-D) materials [20]. 0-D include materials such as quantum dots, whereas 1-D include materials like nanoribbons, nanobelts, nanotubes, nanorods, and nanowires. 2-D materials are nanodisks, nanowalls, nanosheets, nanoplates, and nanoprisms, whereas the 3-D materials are nanoflowers, nanopillars, nanocones, nanocoils, and nanoballs. As per the European Commission, a nanomaterial is referred to as an incidental, natural, or fabricated material consisting of particles, in an unbound state or as an agglomerate or as an aggregate in which 50% or greater of the particles in the number size distribution, and one or more external dimensions in the size range of 1–100 nm [21]. Even though there are at present several types of nanomaterials, it is anticipated that a variety of advanced forms will be developed in the coming years. On the other hand, according to the construction, the US Environmental Protection Agency (EPA) categorized the nanomaterials as (a) carbon-based, (b) composites (combining nanoparticles using other nanoparticles or with bigger, bulk-type material), (c) dendrimers, and (d) metal-based [22]. The different classifications, as well as the extensive range of applications, of the nanomaterials are presented in Figure 1.

The carbon-based NMs have received increased consideration in the engineering and scientific communities [23] and these nanomaterials generally take the shape of tubes, sheets, ellipsoids, hollow spheres, or nanoparticles. The carbon nanotubes (CNTs) are typically manufactured by chemical vapor deposition (CVD) of graphite or by the arc discharge technique [24]. The CNTs are regarded as the stiffest and most robust materials, and the chain of continuous covalent carbon–carbon bonding enables this nanomaterial to become an exceptionally strong material [25]. Graphene is a carbon-based NM arranged in a two-dimensional (2-D) layer of carbon atoms having sp2 hybridization, which are connected in a hexagonal lattice structure [26]. It has outstanding electric and heat conductivity together with optical transparency in the visible and infrared range. Furthermore, due to graphene’s properties such as robustness, high flexibility, and the ability to bind with other elements (for example, metals and gases), this nanomaterial is an extremely attractive option for numerous utilizations [27]. Reduced-graphene oxide (r-GO), graphene quantum dots (GQD), graphene oxide (GO), graphene nanosheets, multilayer graphene, and few-layer graphene are considered to be the graphene-related materials, and all the aforestated nanomaterials could be included in the graphene family materials. Dendrimers are distinct, multivalent molecules possessing branched nanometer size structures. The dendrimer surface has numerous chain ends and might demonstrate variations in shape, size, and adaptability to other elements [28]. Additionally, three-dimensional (3-D) dendrimers consist of inner cavities where various particles could be set for numerous utilizations in materials along with biological sciences [28]. The dendrimers, being nanometric in size, have the ability to non-specifically interact with different cells as well as cellular components manifesting harmful consequences [29]. The metal-based nanomaterials include nanometallic oxides (for example, iron oxide, titanium dioxide, and zinc oxide), nanosilver, nanogold, and quantum dots [30]. Quantum dots (QDs) are considered to be fluorescent semiconductors in the range of 2–10 nm [31]. Semiconductor QDs have attracted substantial research attention due to their better optical properties as well as extensive application in biological and biomedical studies [32].

## 3. Nanomaterials and the Aqueous Environment

The performance of nanomaterials in several aqueous environments inclusive of ocean, river, lake, and wetlands has been explored in the last few years. In order to launch an environmentally benign economy, it has turned out to be a collective concern of environmental research organizations to look for ecofriendly remediating agents for the purpose of restoring the contaminated soil as well as water bodies [33,34,35]. Several recent studies are conducted on electro membrane (separation) technologies for the removal of charged components from solutions such as for producing fresh water from brackish water [36,37,38,39]. Presently, the nanomaterials are generally utilized for the sewage treatment, and the nano-photocatalytic technology is also frequently employed for oxidizing numerous organic compounds existing in water by developing the hydroxide ions as well as superoxide ions with strong oxidation as well as superior activity. Currently, different nanostructured materials are also used in capacitive deionization (CDI), an emerging technology for water treatment.

**Figure 2:**
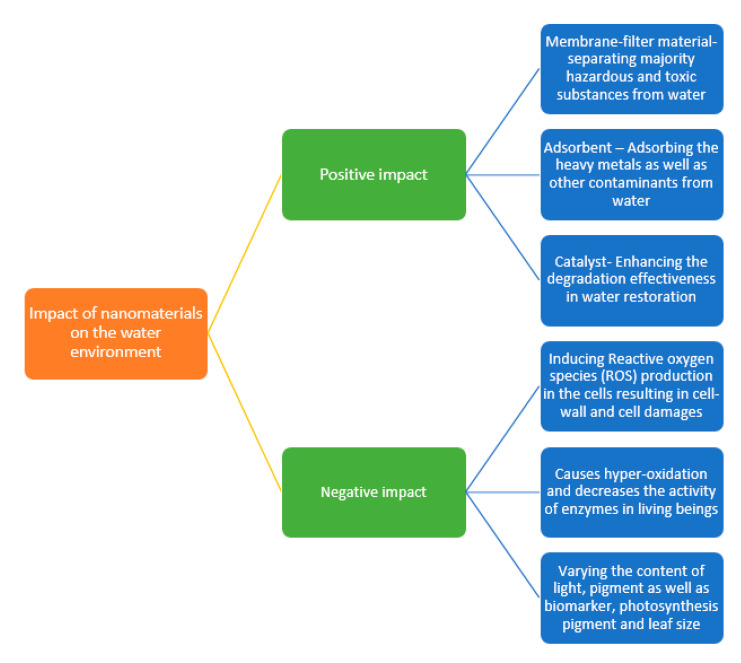
Impact of nanomaterials on the water environment.

The nanomaterials show different behaviors in aqueous environments, in which certain nanomaterials demonstrated the better potential for the remediation of water contamination. The relationship between the water environment and nanomaterials are presented in Figure 2.

There also exists nanofiltration (NF) membrane technology, which utilizes the pressure-driven membranes for separating the substances present in sewage [40]. Furthermore, certain nanomaterials possess an evident adsorption effect on organics or metal ions because of the hydroxyl groups, which are present on the nanoparticle surface, binding to some cations. For example, the carbon nanoparticles, relative to the conventional sewage treatment reagents, demonstrate superior stability in alkaline or acidic conditions and show a huge specific surface area (SSA), particular binding sites, and porous structure. Consequently, the usage of nanomaterials for the treatment of wastewater overcomes the insufficiency of conventional technology and also demonstrates excellent remediation performance [41].

## 4. Application of Nanomaterials in the Aqueous Environment

Nanomaterials like nanoscale graphene, carbon nanotubes, silver nanoparticles, ZnO nanoparticles, TiO_2_ nanoparticles, nanofiber materials, nano-zerovalent iron (nZVI), and NF membrane materials are very beneficial for the remediation of water contamination [14]. The commonly utilized manufacturing techniques, operating principles as well as contamination remediation of the aforestated nanomaterials are presented in Table 1.

Mechanical milling is a nanomaterial manufacturing technique that was developed during the 1970s, as a manufacturing process for fabricating advanced alloys as well as phase mixtures using powder particles [42]. The aforestated technique can overpower the quantity restrictions for nanocrystalline fabrication, and hence the nanocrystalline powders could be manufactured in a huge scale. A significant benefit is that this can function at lower temperatures, and hence the grains formed could develop very slowly. In the case of the plasma process, it could be categorized into two categories, namely plasma spray synthesis and microwave plasma process. The benefits of this technique are the capability of developing unagglomerated particles, narrow particle size distribution, and higher production rates. In the method chemical vapor deposition (CVD), chemical reactions between the gaseous precursor and substrate surface are activated for depositing a thin solid film onto the substrate. The aforestated method is an extensively used material-processing technology because of its ease of scale-up, high production yields and low set-up cost. Laser ablation is a manufacturing technique consisting of two crucial elements: laser beam having an optical focusing system and feeding apparatus. The laser ablation technique is not commonly applied, particularly at a huge-scale, because of its lower yield and increased operational cost. Centrifugal spinning technology can be used to fabricate ultrafine fibers and nanoscale fibers using the centrifugal force [43]. A solution blow spinning method uses elements of both melt blowing ad electrospinning technologies as a substitute method for developing nanofibers.

In the subsequent section, we discuss the positive impact of nanomaterials such as graphene, CNTs, silver nanoparticles, ZnO nanoparticles, TiO_2_ nanoparticles, and nZVI in the aqueous environment.

### 4.1. Adsorbents

Adsorption is considered to be an effective as well as a simple water treatment technique for separating different contaminants present in water sources, inclusive of organic dyes, heavy metal ions, phenols, and so on [49]. The separation efficiency of an adsorption process depends upon the adsorption ability of the adsorbent. Alternatively stated, the surface area exposed and the number of active sites of the adsorbent are very important for the contaminant adsorption. Consequently, nanomaterials appear as an outstanding adsorbent due to its high surface area together with effective active sites [41]. Carbon-based nanomaterials are the most commonly used nano adsorbents, because of their plentiful availability, superior adsorption capacities, high chemical stability, and thermal stability. In the subsequent section, we discuss the usage of nanomaterials in the adsorption of dyes, heavy metal ions, surfactants, pharmaceuticals, personal care products, organic contaminants, phenols, and other toxic contaminants. Table 2 presents a summary of the removal of different contaminants by some of the nanoadsorbents.

#### 4.1.1. Adsorption of Dyes

Currently, dyes perform an important role in paint, textiles, as well as pigment manufacture industries, and no less than 100,000 various types of commercial dyes are available. The effluents of organic dyes and nitroarenes are recognized as a new hazard because of the fact that the undesirable dyes, as well as nitro compounds, are harmful to humans, flora, and fauna [62]. Selective and efficient separation of organic dye from aqueous systems is a persistent universal concern for both potable water as well as wastewater purification. In recent times, several researchers are exploring the utilization of nanomaterials in the remediation of water.

##### Carbon-Based Nanomaterials for the Adsorption of Dyes

**Figure 3:**
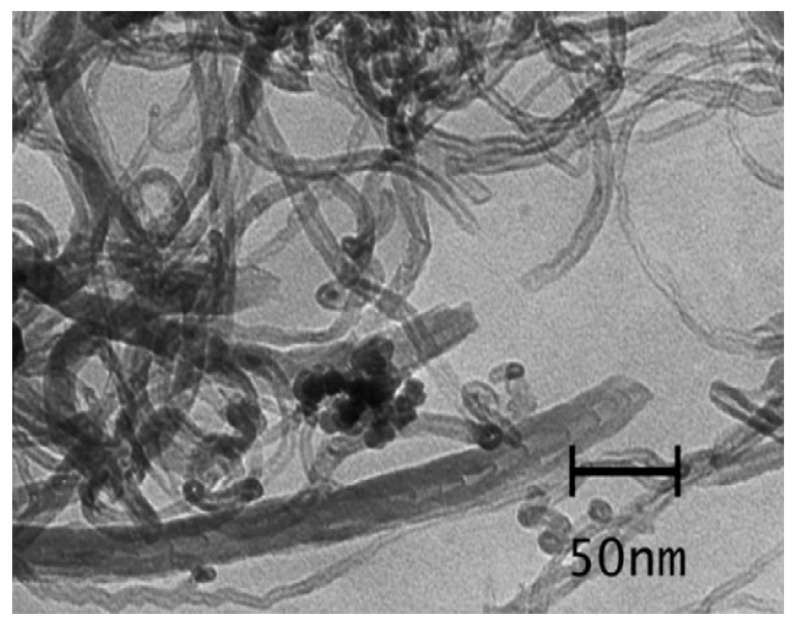
TEM images of magnetic-modified multiwalled carbon nanotubes (CNTs). Reproduced from Ref [61], with permission from Elsevier, 2011.

Studies using CNTs for the removal of dyes started in the year 2004, Fugetsu et al. [63] examined the adsorption of eosin bluish, ethidium bromide, acridine orange, and orange G as typical dyes utilizing the carbon nanotubes. Research carried out by Wang et al. [53] showed high adsorption ability for cationic dye methylene blue relative to the anionic acid red 183 at 10 ppm initial concentration and 0.2 g/L adsorbent dosage at temperature 298 K. For the system with a single dye, the multiwalled CNTs contributed the maximal adsorption capabilities of methylene blue and acid red 183 at 59.70 and 45.20 mg/g, respectively. The solution pH in all the entire tests was retained at a pH value of 6.0. For the system with two dyes, a synergistic effect because of the electronic attraction among the two dyes was noted at less acid red 183 concentration (10.0 mg/L), which promoted the adsorption of two dyes on the multiwalled CNTs. Additional examination proved that the methylene blue planar structure contributed face-to-face conformation that is advantageous for pi–pi bond interactions between the CNTs and methylene blue chromophore. It can be observed that the structure as well as the surface charge of dyes contributed significant roles in the adsorption abilities of carbon nanotubes for various dyes: carbon nanotubes separated the anionic dyes having the planar structure maximum proficiently. Some scientists have tried to develop a magnetic nanocomposite with carbon nanotubes, for allowing efficient separation. Magnetic separation was researched thoroughly by Madrakian and team, utilizing nano-Fe_3_O_4_ composited with carbon nanotubes by means of encapsulation [61]. Contact time has been investigated in order to find the optimum adsorption conditions. Figure 3 is the TEM image of magnetic-modified multiwalled CNTs. The optimal pH for eliminating the entire inspected cationic dyes from aqueous solutions was noted to be 7. This experimental data had been examined using the Langmuir model. The adsorption ability of this nanocomposite with Janus green and methylene blue were noted to be 250 mg/g and 48.1 mg/g, respectively.

**Figure 4:**
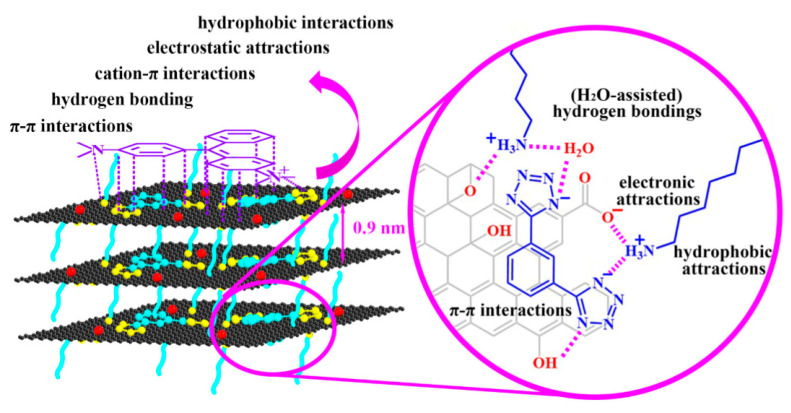
Schematic representation of the non-covalent interactions between graphene oxide, 1-OA, and malachite green (MG). Reproduced from [64], with permission from Elsevier, 2018.

Additionally, the graphene-based nanomaterials can be effectively used for the adsorption of dyes from wastewater. An increased surface to weight ratio along with the superior chemical stability enables the graphene-based nanomaterial to be a potential candidate for the adsorption of inorganic and organic contaminants from aqueous solution. Different organic molecules functionalized graphene oxide nanomaterials by means of non-covalent forces have previously been prepared and utilized in the water treatment. In a research conducted by Lv et al. [64], the team manufactured 1-OA (octadecylamine (OA) as well as tetrazolyl derivative (1)) modified graphene oxide (GO/1-OA) and the adsorbent for eliminating the dyes, bisphenol A (BPA), ciprofloxacin (CIP), and copper ions (Cu^2+^) in their single system, and also to simultaneously coadsorb their quaternary, ternary, and binary contaminants mixture. The schematic representation of the synergistic non-covalent interactions between graphene oxide, 1-OA and malachite green (MG) is shown in Figure 4. Gelatin functionalized carbon-based materials are also examined for water contaminant adsorption [65]. Gelatin reinforcement using nanomaterials (like GO, CNTs, etc.) could enhance the mechanical properties and reduce its rate of degradation because of its superior hydrothermal stability.

##### Metal and Metal-Oxide-Based Nanomaterials for the Adsorption of Dyes

**Figure 5:**
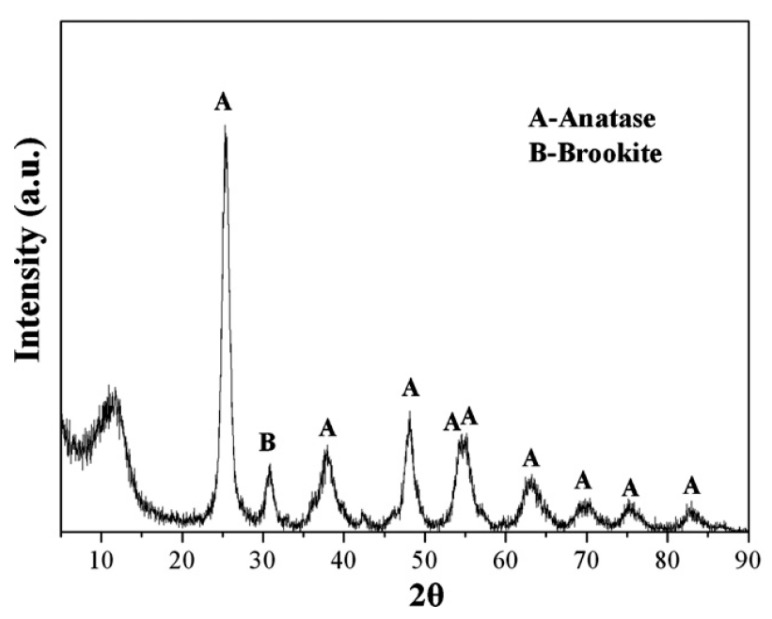
The XRD pattern of the synthesized titanium dioxide nanoparticle. Reproduced from ref [58], with permission from Elsevier, 2009.

The zinc oxide nanoparticle is a high performance, top-end fine inorganic product that could be utilized in several areas like pharmaceuticals, optics, electronics, chemicals, ceramics, etc. Furthermore, zinc oxide nanoparticles could also be used as a water body repair material. Under the ultraviolet (UV) radiation condition the zinc oxide nanoparticles have the capability to diminish the toxic dye MB by almost 97% (initial concentration of MB—10 ppm) [66]. As compared to single zinc oxide NPs, the zinc oxide NPs decorated with other materials have the capability to improve the water purification capability of these nanomaterials. With an efficient nano-titanium dioxide adsorbent, it is probable that dyes can be entirely adsorbed, removing the photocatalytic phase dependence for the dye separation. To reduce the photocatalysis dependency, the nano-titanium dioxide structure can be adjusted to enhance the number of adsorption sites [67]. With the utilization of titanium butoxide, Belessi and team prepared an extremely nano-titanium dioxide, which had the ability for adsorbing reactive red 198 entirely within 10 min, thereby accomplishing an adsorption capacity of 86.96 mg/g [58]. The XRD pattern of the prepared titanium dioxide nanoparticle is presented in Figure 5. At 30 °C temperature, the maximal monolayer adsorption capability attained from the Langmuir model was almost 87 mg/g (at pH 3). Consequently, the photocatalytic reaction was not necessary for the dye degradation.

Nano-zinc oxide is also been utilized as a nanocomposite along with chitosan for the separation of direct blue 78 and acid black 26 [59]. By the immobilization of nano-zinc oxide on the chitosan surface, equilibrium was accomplished with just 3 min. The adsorption abilities of nano-zinc oxide on direct blue 78 and acid black 26 were 34.48 and 52.63 mg/g, respectively. The equilibrium information was examined using Freundlich, Langmuir, and Tempkin isotherms. The team confirmed that the data for direct blue 78 and acid black 26 followed with Tempkin and Langmuir isotherms, respectively. Malwal et al. [57] reported the increased adsorption efficiency as well as the antibacterial properties of electrospun copper oxide-zinc oxide (CuO-ZnO (CZ)) composite nanofibers. It was noted that the Congo red adsorption onto CuO-ZnO composite nanofibers followed the Langmuir model of adsorption isotherm as well as pseudo-second-order kinetics. The maximal adsorption ability for CuO-ZnO composite nanofibers was found to be 126.40 mg/g, which was relatively significant. Consequently, with a superior adsorption capability and satisfactory antibacterial properties, such nanofibers are really a promising and potential candidate in the upcoming water purification as well as water treatment processes.

Due to the fact that the silver has a greater iodine affinity, the silver nanoparticle is adhered to the CA membrane to efficiently separate the radioactive iodine (3.7 MBq). In the course of a water flow rate of 1.5 mL/s, the silver-based cellulose acetate membrane has the ability to filter out excess 99% of radioactive iodine [68]. Furthermore, there is also the incorporation of silver nanoparticles in activated carbon, which could efficiently separate the methylene blue (MB) [69]. The finest conditions for the removal of MB from silver nanoparticles incorporated with activated carbon are an adsorbent dosage of 250 mg/25 mL, contact time of 120 min, and pH of 10. Under specific conditions, the maximal adsorption ability of silver nanoparticles incorporated with activated carbon for methylene blue was 172.22 mg/mL. The adsorption isothermal equilibrium was properly defined by the Freundlich, Langmuir, and Sips models. It could be noted that the contaminant removal from water by silver nanoparticles is mostly attributed to adsorption.

Noticeably, the adsorption abilities for dyes of the studied nanomaterials are at best comparable with activated carbon, and several are found to be higher. With these superior adsorption abilities, the nanomaterials demonstrate high potential as commercially used adsorbents. Some of the adsorbents demonstrate rapid adsorption rates, thereby enabling them possibly desired adsorbents for increased-capacity dye wastewater treatment.

#### 4.1.2. Adsorption of Heavy Metal Ions

Heavy metals like mercury, copper, zinc, lead, etc., can cause a threat to the health of humans due to the fact that they could be biologically accumulated in the food chain. Heavy metals are considered to be extremely toxic as well as carcinogenic [70].

##### Carbon-Based Nanomaterials for the Adsorption of Heavy Metals

Graphene, as the atom layer of carbon, has good potential in removing the heavy metals from wastewater. Several researchers have utilized the graphene nanomaterials for managing the water environment. The conventional techniques of manufacturing graphene nanomaterials include conversion of the carbon nanotube, chemical oxidation–reduction, and mechanical peeling. Graphene as well as graphene oxide are widely reported for removing the heavy metals from wastewater. In order to efficiently remove the very low heavy metal ion concentrations in water, Yap et al. [71] prepared cysteamine functionalized r-GO by the thiol-ene click reaction and used it for the separation of mercury (Hg(II)). Superior removal ability (169 ± 19 mg/g), high selectivity, as well as enhanced regeneration capability were accomplished. It is certain that the chemical-modification of graphene oxide with various functional groups has illustrated fast adsorption kinetics, enhanced adsorption capability, and increased selectivity in the heavy metal ion preconcentration from wastewater [72].

The solution pH value is recognized as a significant parameter that regulates the adsorption of heavy metal ions to solid particles, which will influence the relative distribution of metal ions as well as the surface potential characteristics of graphene oxide-based nanomaterials, specifically the protonation–deprotonation chemical reactions at various pH values [73]. The sorption ability of various sorts of metal ions on distinct types of graphene oxide-based nanomaterials depends on different factors like surface functional groups, metal ion species, and surface properties of graphene oxide based NMs that are normally influenced by solution pH values [44,74]. In addition, the graphene oxide-based composite materials are broadly researched as advanced adsorbent materials because of their superior effectiveness and increased affinity for different metal ions.

Carbon nanotubes are standard adsorbent for the purification of water, however, some remark on their safety is essential. Normally, the carbon nanotubes are needed in huge volumes for the adsorption of water contaminants of very high concentrations. Therefore, it is required to understand what kind of carbon nanotubes are used and the amount of this nanomaterial being utilized. There are several studies on the application of carbon nanotubes for the heavy metal removal from wastewater. Several researchers have purified as well as functionalized the carbon nanotubes utilizing various approaches [75]. The adsorption of water contaminants alters the characteristics of carbon nanotubes such as functionalities, hydrophobicity, stability, surface energy, surface charge, pore size, as well as pore volume. Engineering carbon nanotubes with suitable surfaces as well as surface charges could significantly increase their efficiency in the heavy metal adsorption process. When utilized with a proper functionalization agent, these nanomaterials can be very efficient purifiers for changing the wastewater into potable water.

**Figure 6:**
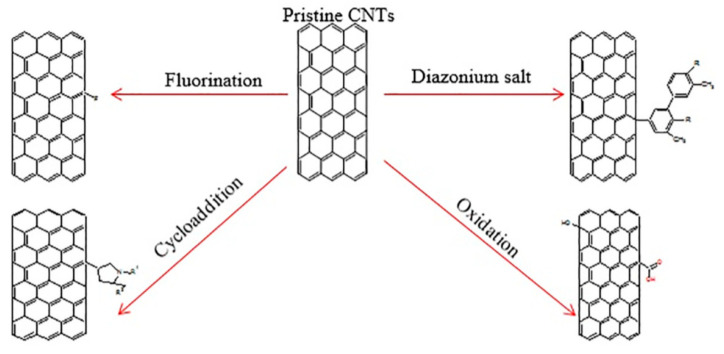
Chemical modifications of carbon nanotubes (CNTs). Reproduced from Reference [78], with permission from Elsevier, 2018.

Several research has confirmed that the advanced multiwalled carbon nanotubes (MWCNTs) fabricated using microwave heating can separate the Zn(II) from water solution, and the rate of removal could attain higher than 99% (initial concentration: 10 mg/L) [76]. Additionally, it can be noted that the functionalized carbon nanotubes can adsorb some heavy metals from water [77]. Various techniques (free radical polymerization, oxidation, reaction with a diazonium salt, fluorination, and cycloaddition), as shown in Figure 6, could be utilized to enhance the reactivity and solubility of carbon nanotubes [78]. Further, there exist some composite materials for the preparation of carbon nanotubes and other substances, which can be utilized to separate contaminants from water. As an example, research has demonstrated that fast adsorption kinetics are noted, and about 65–85% of the bromate ion (initial concentration 5 mg/L) by Fe-CNT nanocomposites were adsorbed at various loadings in just 5 min [79]. The attached Fe ions on the walls/surfaces of nanocomposites can be significant to the adsorption of bromate ion without causing any structural defects to carbon nanotubes.

##### Metal-Based Nanomaterials for Adsorption of Heavy Metals

The nano zerovalent iron (nZVI) particle is regarded as a potential reagent for the fast environmental restoration [80], and these particles have turned out to be a research hot-spot in the area of water remediation. These nanoparticles possess a characteristic core–shell framework with a shell of nonmetallic oxide in a few nanometers thickness enclosing a metal iron core. Additionally, the thin iron oxide shell, through surface complexation and electrostatic interaction, could stimulate the adsorption of contaminants. For the iron-based nanoparticles, surface modification methods help to restrict oxidation of the material along with increasing the stability as well as adsorption capability [81]. nZVI has the drawback of getting oxidized easily, thereby causing reduced adsorption efficiency in sewage. Hence, further research is required in order to overcome this drawback. The utilization of nZVI particles for the remediation of heavy metals from polluted water has restrictions because of its aggregation tendency, lesser durability, and reduced mechanical strength. Thus, L-cysteine (L-cyst) stabilized nano-zerovalent iron (nZVI) particles were prepared by Bagbi et al. [82] by the chemical reduction technique utilizing the L-cyst as a stabilized agent and ferric chloride as a reducer under closed nitrogen atmosphere. A lead batch adsorption analysis was carried out at various pH (from 2 to 7), temperatures (5–45 °C), equilibrium times (5–45 min), initial Pb^2+^ concentrations (10–50 mg/L), and L-cyst-NZVI dosage (1–5 g/L). Nearly 99.9% of lead (Pb^2+^) was separated from 100 mL solution consisting of 50 mg Pb^2+^/L within approximately 25 min, and this can be considered as an economical method for the remediation of lead from water. Ren et al. [83] developed the electrospun polyacrylic acid(PAA)-polyvinyl alcohol(PVA)-nZVI composites successfully and this material accomplished a superior performance in contaminant removal (cadmium) and in mechanical strength improvement for the longstanding filtration process.

#### 4.1.3. Adsorption of the Surfactant

At present, pollution due to the presence of a surfactant is regarded as a severe public environmental issue existing in the aqueous environment [84]. Due to its surface activity features, the surfactants could smoothly diffuse in an aqueous environment and cause eutrophication of water.

##### Carbon-Based Nanomaterials for the Adsorption of Surfactants

The various forms of graphene, namely graphene oxide (GO) as well as reduced-graphene oxide (r-GO), have been utilized for the non-ionic surfactant (TX-100) adsorption by Prediger et al. [85]. The test results confirmed that both r-GO and GO showed the maximum adsorption capacity for the surfactant TX-100 out of the different examined materials.

Gao et al. [86] compared the removal efficiency of three cationic surfactants including hexadecyltrimethylammonium bromide (CTAB), tetradecyl dimethyl benzyl ammonium chloride (TDBAC), and dodecyl dimethyl benzyl ammonium chloride (DDBAC) by different carbon nanotubes. The test results confirmed that maximum removal efficiency of hexadecyltrimethylammonium bromide (initial concentration: 100 mg/L) by pristine multiwalled CNTs with an outer diameter of less than 8 nm is 50.36 ± 0.56%, whereas that by OH-MWCNTs with an outer diameter of less than 8 nm is almost 22.72 ± 0.21%. It was noted that the aromatic cationic surfactants could be easily eliminated by carbon nanotubes, relative to those with no benzene rings because of their powerful π-π interactions. Cheminski et al. [87] synthesized an advanced graphene oxide derivative with phenyl tetraethyleneglycol (PTEG) units attached as stabilizers by arenediazonium salt grafting. The prepared GO-PTEG was used for the removal of cationic (DTAB) and non-ionic (TX-100) surfactant from water. For optimizing the adsorption process, the effect of the parameters such as surfactant concentration, pH, sonication, temperature, time, and nanomaterial concentration was evaluated. The obtained results were noted to be superior to those by graphene oxide and other materials, recommending that the GO-modification enhanced the adsorption process.

##### Other Nanomaterials for the Adsorption of Surfactants

Titanium oxide is a well-known nanomaterial that is used as effective and powerful adsorbents of the removal of surfactants from the contaminated water [88]. Out of the different anionic surfactants, the linear alkyl benzene sulfonate has the greatest consumption and is utilized in huge concentrations in domestic detergents as dishwashing liquids, washing powders, and other domestic cleaners. The work by Abd El-Lateef et al. [89] studied the adsorption of surfactants on zerovalent iron nanoparticles. The nano zerovalent iron particles were effectively prepared, characterized, as well as inspected as an effective adsorbent for the cationic surfactant, the hexadecyl pyridinium chloride surfactant, and the anionic surfactant, sodium dodecylbenzene sulfonate surfactants, from dilute solutions. The thermodynamic parameters obtained demonstrated the spontaneity and easiness of the adsorption process with a promising adsorption extent to the hexadecylpyridinium chloride surfactant compared to the sodium dodecylbenzene sulfonate surfactant.

#### 4.1.4. Adsorption of Pharmaceuticals

The personal care products and pharmaceuticals in surface water are considered to be water contamination sources [90]. The pollution caused by antibiotics also contributes to a severe hazard to the quality of water. Antibiotics possess several potential toxicity risks that could be unsafe to aqueous organisms, and the conventional wastewater treatment techniques have no influence on the separation of certain antibiotics.

##### Carbon-Based Nanomaterials for the Adsorption of Pharmaceuticals

Graphene as well as carbon nanotubes, the commonly utilized carbon-based nanomaterials, are efficient adsorbents for the tetracycline separation. The electrostatic influence could result in strong adsorption of tetracycline to graphene nanomaterials [91]. The existence of phenazopyridine residues in water sources can be dangerous to the human body, henceforth it is extremely important to eliminate these pharmaceuticals from aqueous solutions. In the study by Karimi et al., [92], a magnetic nanocomposite adsorbent reduced graphene–iron oxide was prepared and utilized for the elimination of phenazopyridine contents from wastewater samples. In the finest experimental condition, the reduced graphene–iron oxide nanocomposite demonstrated a phenazopyridine removal efficiency of 91.4% (initial concentration: 15 ppm) in aqueous solution. In work carried out by Zhang et al. [93], nano zirconium carbide was prepared by a preceramic polymers technique, and it was utilized for adsorbing the pharmaceuticals emodin and physcion from aqueous solutions.

##### Metal and Metal-Oxide-Based Nanomaterials for the Adsorption of Pharmaceuticals

It has been observed that titanium dioxide nanoparticles can degrade the personal care products on the basis of their photocatalytic ability [94]. The discharge of non-steroidal anti-inflammatory drugs (NSAIDs) like diclofenac (Dic) naproxen (Nab) and ibuprofen (Ibu) to the aqueous system can lead to severe ecological problems. In the study by Husein et al. [60], green-synthesized copper nanoparticles have been employed as a nano-adsorbent for the separation Dic, Nab, and Ibu from wastewater (initial concentration of solutions 100 mg/L). The maximal removal rates were attained at the conditions: 298 K temperature, 4.5 pH, 10 mg of copper nanoparticles, and at 60 min. At the aforestated conditions, the removal percentage of ibuprofen, naproxen, and diclofenac were found to be 74.40%, 86.90%, and 91.40% respectively. The maximal monolayer adsorption capabilities were found to be 36.0, 33.90, and 33.90 mg/g for diclofenac, naproxen, and ibuprofen respectively. The test results confirmed that the green-synthesized copper nano-adsorbent might be utilized for the separation of the anti-inflammatory drugs from real wastewater effectively. Rahmatinia et al. [95] studied the removal of the metronidazole from aqueous solution using nano-Fe_3_O_4_ by the heterogeneous electro-Fenton process.

#### 4.1.5. Adsorption of Phenol and Other Toxic Contaminants

##### Carbon-Based Nanomaterials for the Adsorption of Phenol and Other Contaminants

There are certain reports on the toxic pollutant degradation in water by carbon nanotubes. For instance, some researchers have established that both phenolic and water molecules are feebly attached to the exterior surface of the original carbon nanotubes, and they could be very intensely adsorbed on the functionalized carbon nanotubes. It has been observed that the phenol binding to CNT–OH was stronger compared to that of water molecules because of the synchronized existence of hydrogen-bonding and π-π stacking in the system [96].

Numerous research [97,98] examined the capability to adsorb organic pollutants by various graphene forms. Jiang et al. [98] analyzed the adsorption of two estrogen pollutants (17α-ethinyl estradiol and 17β-estradiol) by graphene-based nanomaterials, and compared them to other carbon-based nanomaterials such as activated carbon, biochars, and carbon nanotubes in fresh water and in the competition of natural organic matter. Graphene nanomaterials demonstrated better adsorption capability relative to biochars, CNTs, and activated carbons (ACs) under natural organic matter preloading. Graphene-based NMs demonstrated adsorption capability, but the influence of natural organic matter was lesser on graphenes relative to alternate nanomaterials.

As a one-dimensional (1-D) nanomaterial, the carbon nanotubes are very much light in weight and possess several unusual chemical, electrical, and mechanical properties [99,100]. CNTs have developed as the principal NM for the water purification application. This nanomaterial has the ability to separate almost all three kinds of contaminants, i.e., biological, inorganic, and organic contaminants [101]. This is due to the features of CNTs such as high chemical reactivity, a greater aspect ratio, a huge surface area, together with lesser cost as well as energy. Additionally, the adsorption of different organic water contaminants like tannic acid and humic acid changes the properties of CNT and enhances its stability in the environment. The contaminants (for example, biological, inorganic, and organic) must be separated in a way that the properties of CNT will not be altered.

##### Metal and Metal-Oxide-Based Nanomaterials for the Adsorption of Other Contaminants

It was noted that the titanium dioxide nanoparticles could be employed as an adsorbent for removing the disinfection byproducts and natural organic matter from potable water [102]. Stephanie et al. [102] conducted bottle point isotherm tests using raw water from two water treatment plants adjusted to pH 4–8 and dosed with titanium dioxide nanoparticles. The dissolved organic carbon results were in agreement with the modified Freundlich model. The test results illustrated that the pH influenced the separation of natural organic matter from the water surface by titanium dioxide nanoparticles.

The silver (Ag) based NPs display durable absorption along with increased catalytic activity in the visible range and received increased consideration because of its possible utilization in the degradation as well as the separation of organic contaminants under the irradiation of visible light, and are utilized as catalysts because of its enhanced selectivity and high reactivity [103].

The nano zerovalent iron (nZVI) particles, with size lesser than 20 nm, are mostly utilized for the treatment of an extensive range of water contaminants, such as azo dyes, adsorbed nitro-aromatic compounds, aromatic chlorinated substances, and heavy metals from industrial wastewater, and organic chlorinated substances from groundwater. These nanomaterials are more commonly applied for the remediation of water because of the properties and efficiency of nZVI in the separation of contaminants along with its production cost efficiency. Maysoon et al. [104] studied the degradation of acidic aqueous solutions of the Acid red 315 azo dye by nanoscale zerovalent iron Fe^0^ and nano zerovalent iron supported on pillared clay. The test results demonstrated that Acid red 315 azo dye solution was entirely eliminated by nanoscale zerovalent iron Fe^0^ at optimum conditions.

### 4.2. Membranes and Filter Materials

The nanostructured membranes, inclusive of nanofibers, nanoparticles, 2-dimensional (2-D) layer materials, and numerous nanomaterial composites, demonstrate amazing permeation properties and some extra properties (photodegradation, antibacterial, antifouling, etc.), thereby introducing a new path for extremely selective membranes for water purification application [105]. For the application of nanomaterials in membranes for contaminant separation, the nanomaterials can be categorized into two: carbon-based nanomaterials and metal as well as metal oxide-based nanomaterials.

#### 4.2.1. Carbon-Based Nanomaterials for Membrane Applications

Different nanomaterials such as carbon nanotubes and graphenes are noted to have extensive application in membranes and filter materials [106]. The functionalized nonpolar CNT interior offers a robust attraction to polar water molecules and also rejects the salt as well as the pollutant. The aforestated characteristic along with low energy usage and the self-cleaning and antifouling function has turned out that the CNT membranes are an amazing substitute to conventional water treatment techniques. CNTs are common as a separate membrane itself known as the vertically aligned-carbon nanotube membrane. The mixed matrix CNT membrane can be fabricated by doping these nanomaterials into the prevailing polymer membranes such as ultrafiltration, nanofiltration, and reverse osmosis for the improved separation process. Thus, scientists generally categorize the CNT membrane as ultrafiltration (UF), nanofiltration (NF), reverse osmosis, and nano-enhanced membranes. Additionally, engineered carbon nanotubes are making outstanding promises in water purification applications. The engineered carbon nanotubes with suitable functionalities function as a point of attachments where diverse natural water constituents could anchor.

**Figure 7:**
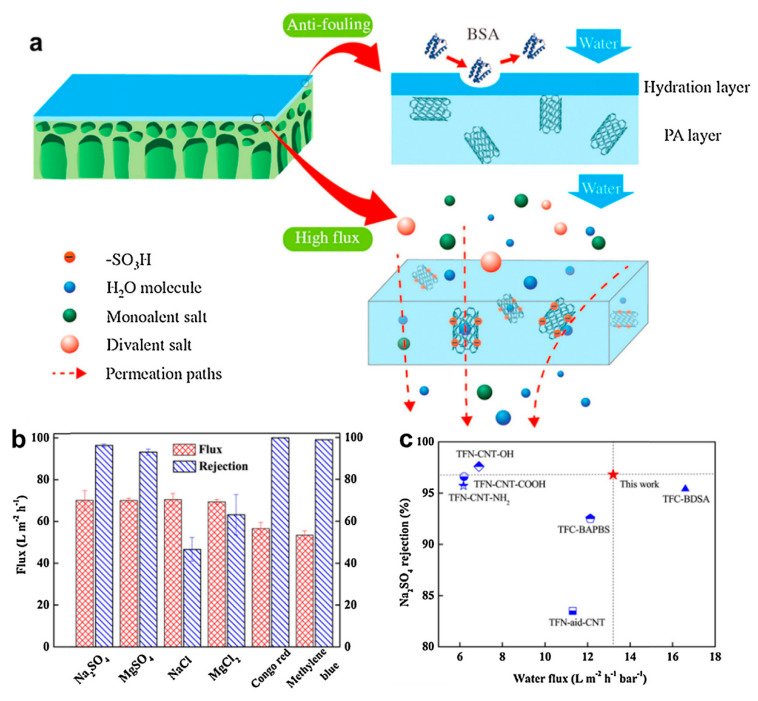
Sulfonated multiwalled carbon nanotube (MWCNT) assisted thin-film nanocomposite (TFN) membrane. (**a**) Representation of the mechanism of membrane separation. (**b**) The separation efficiency of the 0.01% TFN membrane for various salts and dyes at 0.6 MPa and 25 °C. (**c**) Comparing the work results with other sulfonated TFC membranes or the CNT–polymer composite membranes. Reproduced from [109], with permission from Elsevier, 2017.

In a work by Wang et al. [107], the recent advancements made in enhancing the stability of graphene oxide-based membranes in water were properly analyzed. Liang et al. [108] prepared an advanced graphene-based NF membrane having selective laminar nanochannels by filtrating the magnesium silicate nanoparticle modified r-GO nanosheets on a polyacrylonitrile membrane [108]. This composite membrane showed superior selectivity for small molecule separation from water. Both the electrostatic interaction and physical sieving contribute to the rejection. Advanced thin-film nanocomposite (TFN) membranes were synthesized by a standard interfacial polymerization process, with the addition of sulfonated MWCNT into poly(piperazine amide) TFN membranes, Figure 7 [109]. By the optimization of the carbon nanotube content, the salt rejections and water flux of the TFN membranes were freely adjusted for getting the maximum separation efficiency.

In a work by Zdarta et al. [110], reduced-graphene oxide was manufactured by a photocatalytic reduction technique, and reduced-graphene oxide/G–C_3_N_4_ hybrid film was fabricated using a vacuum filtration as well as weak ultraviolet irradiation. The aforementioned film possesses a superior rejection reaction against some salt ions like Na^+^ in a specific quantity of water, and hence the graphene functional film possesses the capability to be the succeeding generation separation film. The entire aforementioned results from different studies demonstrated that the graphene nanomaterials are potential candidates for the remediation of water pollution. Additionally, it can be noted that the graphene nanomaterial might also be transformed in the environment, shifting their capability to adsorb the harmful contaminants. It was also illustrated that black Fe(II) could be utilized as an environmentally benign mild reducing agent of graphene nanomaterials [111], which could remarkably enhance the efficiency of graphene nanomaterials and stabilize the chemical properties of these materials in water.

#### 4.2.2. Metal and Metal-Oxide-Based Nanomaterials for Membrane Applications

Together with the increase in separation efficiency, some of the properties are added to the nanoparticle-related composite membranes, like catalytic, antibacterial, and antifouling properties. Normally, the nanoparticle added for these reasons might consist of hydrophilic metal oxide nanoparticles (e.g., zeolites, TiO_2_, and Al_2_O_3_), a photocatalytic nanoparticle, and antimicrobial nanoparticles. In addition, the thermal as well as mechanical stability of polymer membranes can be increased by inorganic nanoparticles through dropping the adverse impacts of membrane compaction as well as heating [112]. The same as polymer membranes, the ceramic membranes are also modified by the nanoparticle, to improve the efficiency.

Closely packed nanoparticle monolayers, self-assembled from dodecane-thiol-ligated gold nanocrystals, have recently been demonstrated to develop a free-standing strong membrane [113]. These monolayers of a 5.0 nm diameter gold nanocrystal showed exceptional efficiency with filtration coefficients almost 10^−6^ m/(s kPa) for water, which is approximately 100 times higher than in standard polymer-based NF systems. Furthermore, a strong size as well as charge selectivity for different dyes and other molecules with cross-sections less than 1.60 nm at less pressure has been accomplished, with almost 45–60 percent rejection of charged molecules relative to approximately 10.0 percent while uncharged.

### 4.3. Photocatalytic Degradation Application

Environmental decontamination of harmful organic contaminants on catalytic reduction has received increased research attention these days [114]. The release of untreated effluents into the aqueous environment can cause undesirable changes in the environmental balance. Considering the catalytic application of nanomaterials in water treatment, the nanomaterials can be categorized into two: carbon-based nanomaterials and metal as well as metal oxide-based nanomaterials.

#### 4.3.1. Carbon-Based Nanomaterials for Photocatalytic Applications

The contributions from carbon-based nanomaterials to advanced photocatalytic utilization in environmental pollutant treatment such as sorbents, high-flux membranes, depth filters, and pollution prevention strategies have been critically evaluated [115]. In research performed by Alvarez et al. [116], the team introduced a photoactive C_60_ amino-fullerene immobilized on silica gel for producing an amino-C_60_-silica photocatalyst. The aforestated catalyst showed improved oxidation of pharmaceutical contaminants like cimetidine and ranitidine, and MS-2 bacteriophage inactivation in visible-light irradiation, relative to the C_60_ aminofullerene on its own.

The combination of graphene/graphene oxide/reduced-graphene oxide with semiconductor nanomaterials and magnetic Fe_3_O_4_ for the organic contaminants treatment has received huge research interests because of the aggregation as well as quick hole-electron pair recombination of photocatalytic semiconductor nanomaterials, as well as the special properties of graphene/graphene oxide/reduced-graphene oxide [117,118]. Additionally, the multicomponent graphene/graphene oxide/reduced-graphene oxide-based hybrid nanocomposites are also used for photocatalytic degradation as well as the adsorption of organic contaminants, mostly, organic dyes. In a study carried out by Shin et al. [119], the team prepared polyaniline/graphene nanocomposites that photocatalytically degraded the Rose Bengal dye under the visible light irradiation. The greater photocatalytic ability relative to pristine polyaniline can be due to the existence of graphene sheets in polyaniline/graphene nanocomposites that improved photogenerated hole-electron pairs charge separation.

#### 4.3.2. Metal and Metal-Oxide-Based Nanomaterials for Photocatalytic Applications

Considering the textile mills as an illustration, the majority chemicals in these mills are difficult to be degraded as soon as these chemicals reach the water, which will extremely contaminate the aqueous system and impair the well-being of the aqueous environment. According to some reports, the titanium dioxide semiconductor photocatalyst has the ability to degrade certain chemicals like dye in water [120]. Nano-TiO_2_ is extensively utilized as a photocatalyst for degrading dye under ultraviolet radiation. Titanium dioxide nanoparticles have exclusive photocatalytic properties like photochemical corrosion, alkali and acid resistance and non-toxic characteristics, which make these nanoparticles the most favorable photocatalyst. Under the activity of light, the titanium dioxide nanoparticles could be activated and produce free radicals possessing high catalytic activity, which can develop stronger photo-oxidation as well as reduction capability, and catalyze the photodecomposition of numerous organic substances like methanal and certain inorganic substances. As a result, research on the utilization of titanium dioxide NPs in the treatment of wastewater are of excessive progressive significance to the management of the water environment.

**Figure 8:**
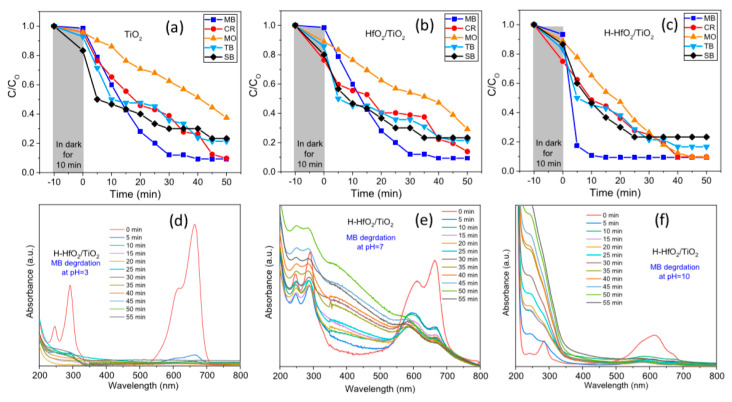
(**a**–**c**) Degradation of 5 diverse dyes (solo-chrome black (SB), thymol blue (TB), cresol red (CR), methyl blue (MB), and methyl orange (MO)) utilizing titanium dioxide (TiO_2_), hafnium oxide (HfO_2_)/TiO_2_, and hydrogenated HfO_2_ doped TiO_2_ (H-HfO_2_/TiO_2_) at pH 7. (**d**–**f**) The degradation of MB dye at various pH employing the H-HfO_2_/TiO_2_. Reproduced from reference [120], with permission from Elsevier, 2018.

The aqueous solutions of different dyes such as solo-chrome black (SB), thymol blue (TB), cresol red (CR), methyl blue (MB), and methyl orange (MO; concentrations: 20 μM) have been photocatalytically degraded utilizing the prepared materials as the catalyst for almost 50 min, as presented in Figure 8a–c. Out of the different dyes, the methyl blue was degraded well by all the three catalysts (titanium dioxide (TiO_2_), hafnium oxide (HfO_2_)/TiO_2_, and hydrogenated HfO_2_ doped TiO_2_ (H-HfO_2_/TiO_2_)), and the shortest duration noted for hydrogenated HfO_2_ doped titanium dioxide with 90% in approximately 10 min. Methyl blue demonstrated strong absorbance at 292 nm and 664 nm with a shoulder peak at 245 nm and 614 nm, as shown in Figure 8d–f.

Alkaykh et al. [121] prepared the nanophotocatalyst MnTiO_3_ powders using the sol–gel method. Of MnTiO_3_ 0.005 g was added into the 50.0 mL methylene blue aqueous solution. The dye solutions had been then centrifuged for 10 min at 4000 rpm. Adsorption kinetic studies were performed by agitating 50 mL of the methylene blue solution with 0.005 g of the adsorbent. The photodegradation was noted to be comparatively greater utilizing a lesser amount (0.005 g) of the semiconductor, obtaining a rate of 70.0% and 75.0% after 240 min for pure MnTiO_3_ and mixed MnTiO_3_/TiO_2_ nanocatalysts. The kinetic model of methylene blue photocatalytic degradation followed pseudo-1st-order kinetic with an elevated correlation coefficient value. The results obtained from this study underlined the utilization of efficient and economical MnTiO_3_ photocatalyst for the decomposition of contaminants in water under natural sunlight.

The titanium dioxide nanoparticles consolidated with carbon materials demonstrated increased solar radiation activity, photocatalytic activity, and these materials are not difficult to separate. Wang et al. [122] demonstrated that the silver based TiO_2_ composite nanotubes showed improved photocatalytic activity in the decomposition of Rhodamine B solution, relative to the bare titanium dioxide nanoparticles. Based on the water/oil phase separation, the silver-titanium dioxide composite nanotubes have been manufactured by the electrospinning (ES) technique with numerous active sites on the exterior, which can increase the photocatalytic ability.

**Figure 9:**
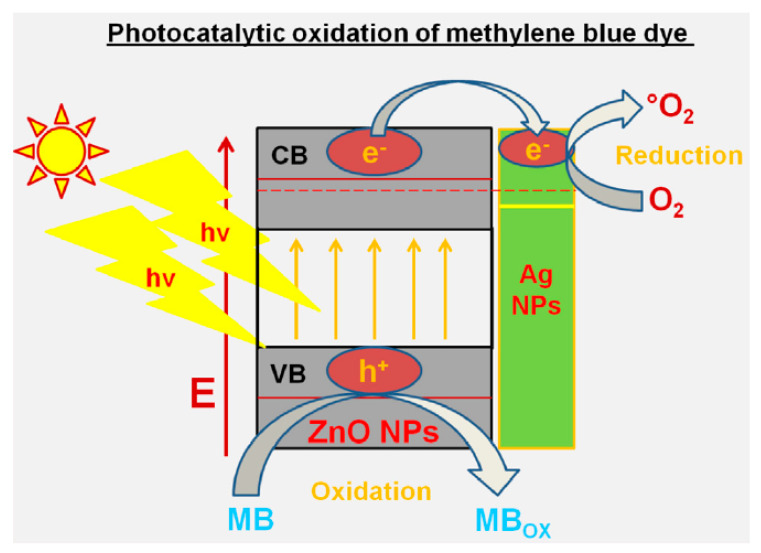
Diagrammatic portrayal of photocatalytic oxidation of the methylene blue (MB) dye at the aminosilicate sol–gel supported silver nanoparticles. Reproduced from reference [123], with permission from Elsevier, 2018.

The zinc oxide nanoparticle demonstrated promising environmental remediation as well as water purification processes by means of the synergic photocatalytic activity by improved oxidation–reduction processes. The diagrammatic portrayal of photocatalytic oxidation of the MB dye at the aminosilicate sol–gel supported silver nanoparticles is shown in Figure 9.

**Figure 10:**
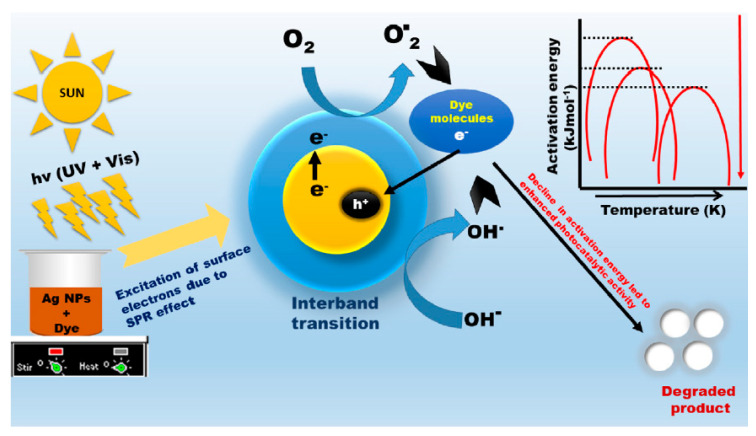
Diagrammatic representation for the suggested mechanism of photocatalytic dye degradation by biogenic silver nanoparticles and correspondingly explaining the activation energy role. Reproduced from Reference [124], with permission from Elsevier, 2019.

Considering the significance of an effective system for photocatalytic utilization, biogenic silver nanoparticles were developed by Singh et al. [124] to examine their photocatalytic efficiency for the separation of two commercial dyes (e.g., reactive blue (RB19) and reactive yellow 186 (RY186); initial concentration: 30 ppm) from water. The bioreduced silver nanoparticles demonstrated a photocatalytic degradation potential of 86% and 88% for RY186 and RB19, respectively (at 180 min). Figure 10 is the diagrammatic representation of the suggested mechanism of photocatalytic dye degradation by biogenic silver nanoparticles and also explaining the activation energy role. Gallo et al. [125] proposed an advanced preparation technique of bimetallic nanosized zerovalent silver/iron (nZVSI), and this nanoparticle demonstrated excellent reactivity, having the ability to degrade the bromophenol blue with appropriate kinetics and consuming a lower amount of material. Furthermore, this bimetallic NM considerably outstripped the nanosized zerovalent silver (nZVS) as well as nZVI attained through an identical procedure.

### 4.4. Microbial Decontamination Applications

Different nanomaterials have demonstrated improved effectiveness in the microbial disinfection procedure while these materials are applied appropriately, which increases the recognition of the nanomaterials in water disinfecting processes [126]. In the application of water disinfection, the nanomaterials can be roughly classified into two: carbon-based nanomaterials and metal as well as metal oxide-based nanomaterials. Table 3 presents the application of nanomaterials for the disinfection as well as bacterial control of water.

#### 4.4.1. Carbon-Based Nanomaterials for Water Disinfection

The carbon nanotubes (CNTs), graphenes, and fullerenes are considered to be renowned nanomaterials due to its disinfecting properties against microorganisms [134]. Both multiwalled CNTs and single-walled CNTs demonstrate efficiency because of their exclusive structures as well as antimicrobial mechanisms. In general, the carbon-based nanomaterials display superior capability for inactivating bacteria, enhanced capacity for the adsorption of viral and bacterial spores due to their higher surface area [135]. Gunawan et al. [136] prepared a polyacrylonitrile (PAN) hollow fiber membrane coated using a silver-multiwalled CNT composite. The utilization of the aforestated composite layer on the hollow fiber membrane for disinfecting use proficiently slowed down the development of the biofilm on the surface of the membrane and stopped the bacteria growth in the filtration module. In research performed by Ahmed et al. [137], greater than 80% inactivation of the microbial cell was observed by the usage of a poly-N-vinylcarbazole-single walled CNT nanocomposite (180 μL of bacterial suspensions (10^7^ CFU/mL)).

Graphene as well as graphene oxide nanoparticles contribute huge opportunities in the disinfection process of water in its composite forms with different metal and metal oxide. Reduced-graphene oxide possesses antimicrobial property that restricts the growth of bacteria thereby limiting the development of the biofilm on the surface of the filter [138]. The graphene oxide nanosheets prepared using silver nanoparticles demonstrate powerful antimicrobial activities against *Escherichia coli* and *Staphylococcus aureus*. Incorporation of silver nanoparticles in graphene oxide nanosheets enhances its antimicrobial effectiveness by 45.0% and 25.0% for *Escherichia coli* (initial concentration: 10^6^ cfu/mL) and *Staphylococcus aureus* (initial concentration: 10^6^ cfu/mL), respectively [139]. Further, different research works confirmed the prohibition of nutrient diffusion into microbe cells because of the cell wrapping by graphene sheets, which contributed to the microbial growth inhibition [140,141].

Fullerenes (for example, C_60_ and C_70_) as well as its derivatives are also acknowledged for their disinfecting capabilities [142,143]. The fullerene C_60_ is reported to demonstrate good antimicrobial activities against *Escherichia coli* and *Bacillus subtilis* by oxidative stress. Further, the nanomaterials graphene as well as graphene oxide show prodigious opportunities in water disinfecting processes in the composite forms of these nanomaterials with different metal and metal oxides [144,145]. Reduced-graphene oxide also demonstrates an excellent antimicrobial feature that inhibits the growth of bacteria, thereby delaying the biofilm development on the surface of the filter [146].

#### 4.4.2. Metals and Metal-Oxide-Based Nanomaterials for Water Disinfection

Different metals as well as metal oxides demonstrate good antimicrobial capability in water disinfection. Out of the different metals, the silver ions and silver compounds are commonly used for disinfection purposes because of their superior antimicrobial activity and better germicidal impacts [147]. Silver nanoparticles are extensively integrated into numerous products, partly because of their antimicrobial properties. Zinc oxide nanomaterials also display increased antimicrobial activities against an extensive range of bacteria inclusive of *Escherichia coli* and *Lactobacillus helveticus* [148]. An advanced antibacterial filter was developed by Li et al. [149] for research and a granule material with red mud was used as the raw material. The nano ZnO as a modifier, and a cationic polyelectrolyte layer was effectively prepared. Nano-ZnO-polyelectrolyte diallyldimethylammoniumchloride (PDDA) modified red mud granule material possessed high SSA and numerous adsorption sites, demonstrating superior antibacterial properties. This nano ZnO-polyelectrolyte diallyldimethylammoniumchloride modified red mud granule material is suitable to manufacture, easy to separate, low in cost, and exceptional in performance, which is advantageous to diminish the microbial contamination of water bodies as well as the hazard of disinfection byproducts. Additionally, the titanium dioxide-iron(III) oxide nanocomposite was observed as an efficient microbial disinfectant, which could eliminate *Escherichia coli* by means of photocatalytic activity [132]. Microbial cell inactivation by metals and metal oxide nanomaterials is mostly by cell physical damage.

Thus, it can be noted that nanomaterials have been expansively utilized for removing contaminants present in water due to their extraordinary properties. There still exist some problems that should be overcome for making improved usage of the nanomaterials in the water treatment. Majority of the nanomaterials are unstable and have the tendency to aggregate, thereby diminishing the separation ability. Further, it is typically hard to remove the nanomaterials present in the aqueous solution effectively because of their nanoscale size. The commercially used nanomaterials utilized for contaminant separation on a commercial scale are rare and additional efforts are required for developing commercial nanomaterials. The development of nanomaterials has offered us with a potential substitute to the conventional adsorbents for separating the contaminants.

## 5. Toxicity of Nanomaterials in the Aqueous Environment

Currently, the nanomaterials are progressively as well as broadly utilized in several areas, like materials science, cosmetics, pharmaceuticals, ecology, and so on [150,151]. On the other hand, the utilization of nanomaterials also causes some unsafe effects on the environment and ecology [152,153]. Several nanomaterials are unavoidably discharged into lakes, rivers, and seas. Potable water can ultimately be influenced by these nanomaterials as well. As a result, the pollution of water environments resulting from these nanomaterials might be toxic to aqueous life or human life. Various kinds of nanomaterials or even similar types of nanomaterials at varying sizes have been noted to have an incompatibly harmful effect on certain organisms. There are a huge number of microorganisms available in aqueous environments. Therefore, it is a usual event for nanomaterials to interact with microorganisms once they enter aqueous environments. The function, as well as toxicity, of commonly used nanomaterials in aqueous surroundings are presented in Table 4. In the succeeding section, we discuss the negative impact of NMs such as CNTs, silver nanoparticles, and graphene-based nanomaterials in the aqueous environment.

### 5.1. Cellular Damage/Membrane Damage

The carbon nanotubes are not soluble in water and these nanomaterials are deposited in sediments, which is considered to be harmful to benthic animals in water, and furthermore it influences the transportation of other coexisting contaminants [171]. Several researchers have also examined the toxicity of carbon nanotubes to aqueous organisms. In a study carried out by Zhu et al. [172], it was demonstrated that the single-walled carbon nanotube (SWCNTs) demonstrated a huge influence on the growth of *Artemia salina* in seawater, and with the greater concentration of single-walled carbon nanotubes greater larval mortality was observed. Parks et al. [173] analyzed the bioaccumulation, bioavailability, and toxicity of single-walled CNTs in marine benthic organisms (*Leptocheirus plumulosus, Americamysis bahia,* and *Ampelisca abdita*). It was demonstrated that the single-walled CNTs were bioaccessible to marine benthic organisms, however, it will never accumulate or give rise to toxicity. The CNTs might get trapped and accumulated in the surface microlayers of the ocean because of the surface tension, as well as viscous properties, of microlayers.

Da Rocha et al. [174] examined the carbon nanotube toxicity to zebrafish. Serotonin (5HT) and dopamine (DA) are important neurotransmitters for behavioral responses and brain functions. The analysis of dopamine and serotonin have been performed in brain samples from zebrafish *Danio rerio* subjected to SWCNT, in addition to examining ectonucleotidases and acetylcholinesterase (AChE) activity. Test results demonstrated that the SWCNT treatment enhanced between 3 and 6-fold the concentration of dopamine and serotonin. In the same way, a substantial drop in acetylcholinesterase activity has been noted in the brains of zebrafish subjected to SWCNT relative to the control groups. Dopaminergic, serotonergic, and cholinergic systems, through dopamine and serotonin levels, and acetylcholinesterase activity, respectively have been influenced by a single-walled carbon nanotube in the brain of zebrafish. Variations in these neurotransmitters could possibly disturb numerous physiologies and behaviors that they regulate.

The carbon nanotubes can vary the oxidation performance of enzymes in water molecules, which might be toxic to microorganisms [175]. In research work conducted by Tabei et al. [176], the toxicity analysis of MWCNT was carried out, and it was demonstrated that this nanomaterial has limited cytotoxicity for already differentiated HL-60 cells and exceptionally high phagocytic activity for undifferentiated HL60 cells. Furthermore, the multiwalled carbon nanotube has specific genotoxicity, which would influence the repair mechanism of DNA. Utilizing the molecular larval examination at aquatic levels, Martinez-Paz et al. [177] demonstrated that the MWCNTs influence the transcription of genes associated with apoptosis.

**Figure 11:**
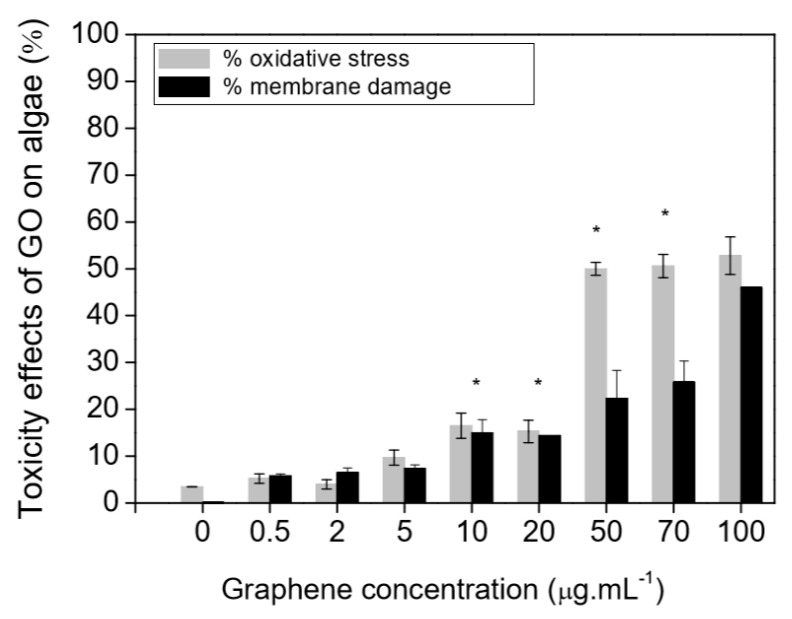
Toxicity effects (oxidative stress and membrane damage) of GO on *R. subcapitata* green alga; evaluated by flow cytometer. Values are mean +SD (N = 3). * statistically significant difference from control (*p* < 0.05). Reproduced from reference [178], with permission from Elsevier, 2015.

**Figure 12:**
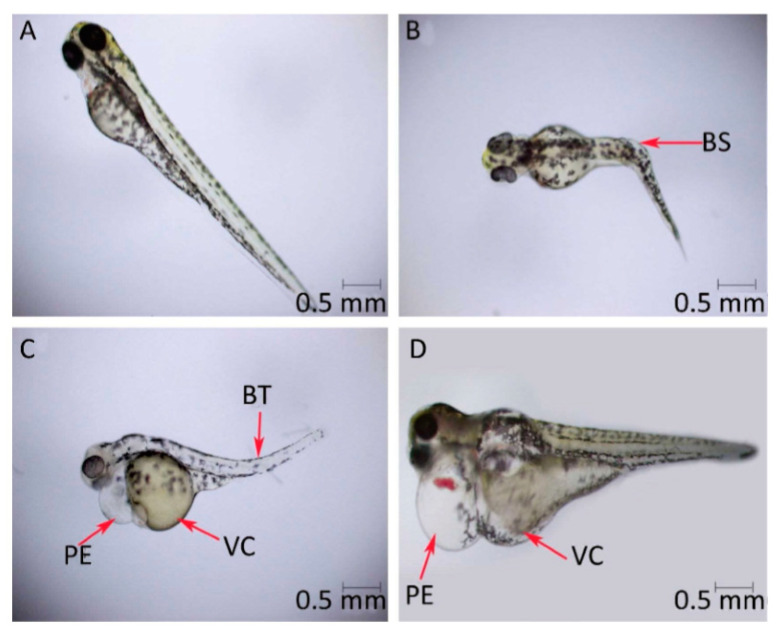
Deformity of zebrafish embryos when exposed to graphene quantum dots of concentration 200 μg/mL, scale bar = 0.5 mm. (**A**) Standard larvae and (**B**–**D**) abnormal larvae. Deformities are specified by red arrows. BT, bent tail; BS, bent spine; VC, vitelline cyst; and PE, pericardial edema. Reproduced from reference [185], with permission from Elsevier, 2015.

Toxicity to marine organisms might also be a significant problem subsequent to the environmental release of graphene-based materials. Graphene oxide is noted to generate toxic effects in terms of reactive oxygen species generation as well as membrane damage in *Raphidocelis subcapitata* green algae [178] and inhibition of cell rates division in *Chlorella vulgari*, a different green alga [179]. Figure 11 is the representation of toxicity effects (oxidative stress and membrane damage) of graphene oxide on *R. subcapitata* green alga; evaluated by the flow cytometer. The probable toxicological consequences in photosynthetic organisms by a functionalized graphene oxide form have correspondingly been examined. Ouyang et al. [180], utilizing the *Dunaliella tertiolecta* green alga confirmed that the internalization of graphene oxide quantum dots has been noted to be 10–80 times greater relative to the nano-graphene oxide resulting in increased oxidative stress, cell permeability, and cell division. Du et al. [181] examined the phytotoxicity of reduced-GO to microalgae *Scenedesmus obliquus* demonstrated that the treatment with reduced-GO inhibited the growth of microalgae. Additionally, reduced-graphene oxide has restrained the levels of chlorophyll a and b in the algal cells. X. Zhang et al. [182] and P. Zhang et al. [183] demonstrated that the burrowing activity of the oligochaete, *Tubifex tubifex,* has been considerably diminished after the graphene oxide exposure. Another research similarly confirmed that the graphene oxide leads to the variation with respect to biochemical performances [184]. For instance, in the polychaete *Diopatra neapolitana* the graphene oxide appeared to change the energy-associated responses and cause cellular damage, in spite of greater activities of antioxidants as well as biotransformation enzymes in individuals subjected to these pollutes [184]. Toxic impacts of graphene quantum dots were also examined in zebrafish embryos that demonstrated that these graphene-based nanomaterials induced larval hyperactivity as well as embryonic malformations [185]. Figure 12 is the representation of the deformity of zebrafish embryos when exposed to graphene quantum dots of a concentration of 200 μg/mL.

It was demonstrated that the silver nanoparticles could lead to negative impacts on environmental flora and fauna, like nematode, alga, fungi, etc. [186,187]. The benthic species perform a substantial role in the function and structure of marine ecosystems. When the silver nanoparticles are discharged from commodities into an aqueous system, the aqueous species, particularly the benthic organisms, can be in danger. An aquatic mesocosm analysis illustrated that silver nanoparticles induced damage to DNA and oxidative damage in *Scrobicularia plana* [188]. These nanomaterials also induce hepatopancreas pathology and the development of early apoptosis of *Mytilus galloprovincialis*.

An investigation was performed with a freshwater bivalve exposed to polyvinyl pyrrolidone coated silver NPs by Liu et al. [162]. In the aforestated study, *Corbicula fluminea* enabled the motion of silver nanoparticles from water to sediment and influenced the fate as well as the transformation of silver nanoparticles. Henceforth, a huge amount of silver accumulated in sediment, causing danger to benthic organisms. The test results confirmed that the silver bioaccumulation caused oxidative damage as well as the interdiction of physiological metabolism in *Corbicula fluminea*. Various detoxification mechanisms were noted in *Corbicula fluminea* at diverse doses of silver nanoparticles. As reported by the bioaccumulation of silver nanoparticles in shells and bodies, the shells are a superior choice for higher silver nanoparticle concentration indicators, while the tissues are increasingly sensitive to low concentration. Silver nanoparticle was not seen in feces of *Corbicula fluminea*, probably because of the fact that the silver NPs had lengthier gut retention time relative to other substances. Further, higher concentrations of silver nanoparticles possible induced the calcospherite disintegration and calcium loss. The aforestated study further provides the knowledge of the interaction between *Corbicula fluminea* and silver nanoparticle, contributing beneficial data on the toxicity and fate of silver NPs occurring in the natural aqueous system.

### 5.2. Other Negative Impacts

Thakkar et al. [189] analyzed the influence of single-walled CNTs on *Dunaliella tertiolecta* and illustrated that enhancing the concentration of SWCNTs boosted the toxic effects on oxidative stress, photosynthesis, and growth. Furthermore, the length, diameter, and structure of carbon nanotubes are also significant factors for the toxicity of these nanomaterials. As the length of carbon nanotubes increased, the toxicity of this nanomaterial will also increase. As an illustration, it was proved that the macrophages would absorb CNTs with a short length, whereas the CNTs with longer lengths will not be absorbed [190]. Jang et al. [191] proved that functionalized MWCNT in the aqueous environments could lessen the lead toxicity in the environment. On the other hand, Kim et al. [192] illustrated that carbon nanotubes could increase copper toxicity. In the real environment, the carbon nanotubes cannot exist unaccompanied, and these nanomaterials possess a limited level of adsorption to other ecological pollutants, so it is not possible to precisely evaluate the harm brought about by carbon nanotubes to the environment.

The degree of toxicity of silver nanoparticles changes with water chemistry as well as characteristics of sediments [193] probably due to the fact that the silver nanoparticles interact with natural water colloids subsequent to their discharge. The aforestated could influence the stability as well as the consequent environmental behavior of both aquatic colloids and silver nanoparticles. Trace metals could be related to colloids and particles, which perform a significant role in the transport of heavy metal, and can be influenced by silver nanoparticles. Tao et al. [163] proved that besides the present metal pollution status, the silver nanoparticles might perform as a coagulant, which could deposit ions as well as colloids consisting of Cd, Pb, Zn, and Cu from the pore water of the sediment, resulting in a reduction in the concentration of heavy metal. Conversely, part of the silver nanoparticles may get stabilized in pore water and develop a silver-mercury amalgam, which might represent a significant type of mercury transformation in the sediment. The aforestated research work has demonstrated that mercury-silver amalgam development can lead to considerable colloidal mercury mobilization to surface water and be transported across the stream to uncontaminated areas. Therefore, the mercury-silver amalgam NPs might remarkably enhance mercury transportation across larger distances, upon their discharge into surface water.

These graphene-related NMs will be certainly discharged into the surroundings in the course of fabrication, transport, utilization as well as disposal. When these materials are discharged into waters, they will have the opportunity to interact with different types of biological and physicochemical factors, as a result leading to substantial negative effects to the surroundings with consequences at the ecological unit level [156]. Several research works have concentrated on the toxicity of graphene-related NMs in the aqueous environment and it was proved that the toxicity of these graphene-based NMs depends on their surface charge, size, shape, exposure concentration, and nature [194] and also on the target species, route of particle administration, medium composition, and the time of exposure [195]. A major problem to be considered during the environmental risk valuation of graphene-based nanomaterials in the aqueous system is indicated by their adsorption abilities. The greater water solubility, as well as the smaller size, of these functionalized GO nanomaterials make these materials exceptionally hard to be removed from wastewater subsequent to the adsorption of heavy metal ions.

## 6. Regulations

Nanotechnology is sensational for the reason that the state-of-the-art of manipulating matter at the nanoscale is in its early stages, and the opportunities to be explored are uncharted as well as extensive. Thus, this novelty results in indescribable and unforeseen dangers. The increasing influence of exposure to different types of nanomaterials will also be subject to inspection utilizing novel legal tools to allocate responsibility due to the fact that exposures will be in combinations that cannot be quantified in places where exposure cannot be controlled and the source of potential harms can stay unknown.

Novel products or technology must undergo wide testing for adversarial health and environmental consequences prior to the introduction. In the study carried out by Kriebel et al. [196], the team defined the preventive codes for ecological decision making as “(1) taking precautionary act in the face of improbability; (2) shifting the burden of proof to the proponents of an action; (3) discovering an extensive range of substitutes to possibly dangerous actions; and (4) enhancing the participation of public in making decisions”. Nevertheless, such codes have not been followed prior to the introduction of nanomaterials, leaving insecurity about the hazards against the benefits of nanomaterials. Although there exists an argument among the research community concerning appropriate safety valuation of nanomaterial exposure for regulation as well as governance, the regulatory and government authorities are operating to implement substantial regulation about nanomaterials. Even though there are arguments among regulatory groups, scientists, and employers regarding the adequate range of hazards, there are no existing standardized approaches for determining as well as characterizing nanomaterials, which makes it extremely hard to establish any suitable evaluation. Except for a few exclusions, there are no precise regulations as regards nanomaterial exposure. Subsequent to several years of serious revisions, European commission newly admitted that the nanomaterials are “difficult to regulate” due to their complexity as well as deficiency of knowledge [197].

## 7. Discussion and Conclusions

This work has reviewed the recent application of nanomaterials in the water treatment for environmental remediation. Additionally, we discussed the negative impacts of nanomaterials in water. We mainly analyzed the impacts of nanomaterials such as carbon nanotubes (CNTs), graphene-based nanomaterials, silver nanoparticles, zinc oxide (ZnO), titanium dioxide (TiO_2_), and nano-zerovalent iron.

From the analysis of different papers, it can be confirmed that the graphene nanomaterials showed good adsorption capacity for the surfactant, antibiotics, and other harmful contaminants. These nanomaterials can also effectively remove the very low concentration heavy metal ion in water. Additionally, they exhibited increased selectivity as well as high regeneration capability. Even though there are numerous issues in the fabrication of graphene oxide-based NMs and their utilization in environmental contamination purification, through the different steps taken by the scientists, it is anticipated that the graphene oxide-based composites could be utilized in the area of wastewater treatment in practical utilization in the upcoming years. Additionally, it was noted that the carbon nanotubes could act as effective purifiers and have the capability for separating biological, inorganic, and organic contaminants from water. Additionally, the functionalized carbon nanotubes have good ability to remove some organic dyes and adsorb some heavy metals present in water. Further, the functionalization with hydroxyl and carbonyl groups of CNTs can be utilized in organic pollutant degradation. Thus it is clear that innovative modified CNT-based materials can function as a prospective material for the water remediation solution. It can be observed that the silver based nanoparticles demonstrate increased catalytic activity in the visible range, durable absorption, and also it has good utilization in the degradation and separation of organic contaminants from water under the irradiation of visible light. Further, these nanomaterials are used as catalysts due to its improved selectivity and high reactivity. Additionally, they can separate the commercial dyes from water. Further, we observed that the zinc oxide nanoparticles have the ability to diminish the toxic dyes present in water. This material showed promising environmental remediation in the aqueous environment through synergic photocatalytic activity. Additionally, the ZnO-based nanofibers are potential candidates in the upcoming water purification and water treatment processes. Further, the zinc oxide-based nanomaterials are used to reduce the microbial contamination of water bodies and the hazard of disinfection byproducts. The examination of different studies confirmed that out of the various nanomaterials, graphene and its derivatives (e.g., reduced graphene oxide, graphene oxide, graphene-based metals, and graphene-based metal oxides) with a huge surface area, increased purity, and outstanding environmental compatibility and selectivity display high absorption capability as they trap electrons, avoiding their recombination. Hence, the graphene-based materials can be best suited for the water remediation application.

When nanomaterials are discharged into waters, they will have the opportunity to interact with different types of biological and physicochemical factors, as a result leading to considerable negative effects to the surroundings with consequences at the ecological unit level. In addition, the carbon nanomaterials possess the ability to interact in a complicated way with the co-occurring pollutants, altering the bioavailability and, thus, their resultant toxicity in either an antagonistic or a synergistic way. Other nanomaterials, like metal-oxide-based nanomaterials, might modulate the harmfulness of copollutants. Additionally, proper research in this field is missing; irrespective of various characterization methods, it is critical to consider the various media in which the nanomaterials are dispersed. It was noted that enhancing the concentration of nanomaterials boosted the toxic effects on oxidative stress and growth of green algae. Scientists are still not sure if certain nanomaterials will form an absolutely new class of non-biodegradable pollutants. In the water environment, the nanomaterial stability depends on the characteristics of nanomaterials (shape, size, concentration, zeta potential, surface chemistry, and hydrophilicity) and the physicochemical parameters of the water environment (presence of organic matter, alkalinity, biochemical oxygen demand, pH, hardness, and ionic strength).

In order to facilitate the accomplishment of the nanomaterial-based water treatment techniques, there still exist numerous milestones that should be reached. Nanomaterials could be utilized in the preparation of nanoadsorbents with distinct surface functionalities that optimally capture non-polar as well as polar contaminants present in water. With regards to the photocatalysis-based water treatment, it is very important to hinder electron-hole recombination in the photocatalyst, and also to substitute the ultraviolet light using the solar visible-light as the major stimulation for the process. This solar visible-light utilization will assure energy proficiency and more widespread usage of the photocatalysis process in the water treatment. For the nanoadsorbents, major obstacles for the utilization are their aggregation affinity and problematic recovery. For addressing the aforestated issues, it can be deposited on the nanofiber substrate. Additionally, in the case of nanofiber membranes, the major issue hindering its commercialization is the absence of accurate and consistent testing of the membranes. The nanofiber membranes should undergo testing over an extended period of time, and under the mechanical, thermal, and chemical conditions normally observed in practical terms and with actual wastewater models. Furthermore, non-toxic materials must be used for the water treatment operations that are considered to be ecologically less challenging. In this respect, the advanced generation of naturally occurring nanomaterials like cellulose-based nanomaterials can be favorable. Additionally, it is important to appropriately design the systems such that they slightly discharge nanomaterials into the surroundings.

By lessening the massive gaps in information about the nature of nanomaterial interactions, we will have appropriate strategies with regards to the applications, processing, as well as regulation of nanomaterials in the coming years. Standard proceedings should be ensured to permit for ecologically benign usage of these nanomaterials and allow for a viable future growth, from industrial utilization to health care as well as environmental approaches. Scientists look forward to optimizing the advantages of nanotechnology potential while controlling and minimizing the hazards.

## Figures and Tables

**Table 1:** Application of various nanomaterials in the remediation of water contamination.

Sl. No.	Nanomaterials	Common Manufacturing Techniques	Mechanism	Remediation	Reference
1	Graphene nanomaterials	Photocatalytic reduction, carbon nanotube (CNT) conversion, chemical oxidation–reduction, mechanical milling, pyrolysis, and plasma synthesis	The porous structures present in the nanomaterial adsorb the contaminants present in water	Dyes and heavy metals	[44]
2	Titanium dioxide (TiO_2_) nanoparticles	Liquid phase technique and gas-phase technique	The ultraviolet (UV) light in light could stimulate the TiO_2_ nanoparticles and develop the free-radicals having high catalytic activity, that could generate a stronger photo-oxidation as well as reduction capacity	Certain inorganic substances and different organic substances like formaldehyde	[45]
3	Zerovalent iron (nZVI)	Reduction	The thin iron oxide core could support the pollutant adsorption by means of the surface complexation and electrostatic interaction.	Azodyes, nitro-aromatic compounds, chlorinated aromatic compounds, heavy metals, and organo-chlorine.	[46]
4	Nanofiber material	Electrohydraulic dynamic (EHD) direct writing, centrifugal jet spinning (CJS), solution blowing, electrospinning (ES), molecular techniques, etc.	Particles having various diameters are filtered using membranes possessing distinct pore sizes.	Different contaminants present in eutrophicated landscape water	[47]
5	Silver nanoparticles	Radiation, electroplating, laser ablation method, photoreduction, and chemical vapor deposition.	By coupling with the bacterial metabolic enzymes, the bacteria will get dried up as well as later die. Superior germicidal performance can be observed in smaller size particles	Role of a water photocatalyst and antibacterial agent	[48]

**Table 2:** Summary of the removal of different contaminants by some of the nanoadsorbents.

Sl. No.	Nanomaterial	Contaminant	Max Adsorption Capacity (mg/g)	Model	Conditions	Important Findings	Reference
1	Single-walled CNT	Cr (VI)	2.35	Langmuir	Cr (VI) C_in_ = 0.2–1.0 mg/L CNT C_in_ = 25–200 mg/L	pH dependent (Optimum = 2.5) and time dependent	[50]
2	Multiwalled CNT	Cr (VI)	1.26	Langmuir	Cr (VI) C_in_ = 0.2–1.0 mg/L CNT C_in_ = 25–200 mg/L	pH dependent (Optimum = 2.5) and time dependent	[50]
3	Single-walled CNT	Ciprofloxacin (CIP)	724	Brouers-Sotolongo	CIP C_in_ = 50 mg/L CNT C_in_ = 100 mg/L	pH dependent (Optimum= 7) and time dependent	[51]
4	Multiwalled CNT	Ciprofloxacin (CIP)	475	Brouers-Sotolongo	CIP C_in_ = 50 mg/L CNT C_in_ = 100 mg/L	pH dependent (Optimum = 7) and time dependent	[51]
5	Single-walled CNT	Oxytetracycline (OXY)	554	Brouers-Sotolongo	OXY C_in_ = 50 mg/L CNT C_in_ = 100 mg/L	pH dependent (Optimum = 7) and time dependent	[52]
6	Multiwalled CNT	Oxytetracycline (OXY)	391	Brouers-Sotolongo	OXY C_in_ = 50 mg/L CNT C_in_ = 100 mg/L	pH dependent (Optimum = 7) and time dependent	[52]
7	Multiwalled CNT	Methylene blue	59.7	Langmuir	MB C_in_ = 5–15 mg/L C_in_ = 0.02 g	pH dependent (Optimum = 6)	[53]
8	Multiwalled CNT	Acid dye	45.2	Langmuir	Acid dye C_in_ = 10–50 mg/L graphene C_in_ = 0.02 g	pH dependent (Optimum = 6)	[53]
9	Graphene	Methylene blue (MB)	204.08	Langmuir	MB C_in_ = 20–120 mg/L graphene C_in_ = 0.02–0.17 g	Adsorption is pH insensitive	[54]
10	Reduced GO/Mg(OH)_2_MgO	Arsenite (As (III))	681	Langmuir	As C_in_ = 1.5 mg/L adsorbent C_in_ = 400 mg/L	Optimum time—360 min	[55]
11	GO/Fe_3_O_4_	Arsenic (V)	5.27 (Langmuir), 1.99 (Freundlich)	Langmuir and Freundlich	Ar C_in_ = 5 mg/L adsorbent C_in_ = 200 mg/L	Temperature dependent	[56]
12	CuO-ZnO nanofibers	Congo red dye	126.4 Langmuir	Langmuir, Freundlich and Temkin	CR C_in_ = 10–90 mg/L nanofibers C_in_ = 2 mg	-	[57]
13	TiO_2_ nanoparticles	Reactive Red 195	87	Langmuir	RR C_in_ = 10–50 mg/L adsorbent C_in_ = 0.02 – 0.2 mg	Followed pseudo-second-order expression	[58]
14	ZnO	Direct blue 78	34.48	Langmuir, and Temkin	DB C_in_ = 50 mg/L adsorbent C_in_ = 0.05 – 0.2 mg	Followed pseudo-second-order kinetics	[59]
15	ZnO	Acid black 26	52.63	Langmuir, and Temkin	AB C_in_ = 50 mg/L adsorbent C_in_ = 0.05 – 0.2 mg	Followed pseudo-second-order kinetics	[59]
16	Copper	Ibuprofen	33.9	Langmuir	Ibu C_in_ = 10–40 mg/L	Adsorption spontaneous and endothermic	[60]
17	Copper	Naproxen	33.9	Langmuir	Nap C_in_ = 10–40 mg/L	Adsorption spontaneous and endothermic	[60]
18	Copper	Diclofenac	36.0	Langmuir	Diclo C_in_ = 10–40 mg/L	Adsorption spontaneous and endothermic	[60]
19	Magnetic-modified multiwalled CNTs	Janus green	250 mg/g	Langmuir	JG C_in_ = 20 mg/L	Optimal pH—7	[61]
20	Magnetic-modified multiwalled CNTs	methylene blue	48.1 mg/g	Langmuir	MB C_in_ = 20 mg/L	Optimal pH—7	[61]

**Table 3:** Application of nanomaterials for the disinfection as well as bacterial control of water.

Sl. No.	Nanomaterials	Microorganisms Removed	Efficiency of Removal	Reference
1	Silver nanomaterials loaded kaolin clay	*Escherichia coli*, *Salmonella* spp.	80% for *Escherichia coli*; 9 % for *Salmonella* spp. (concentration: 0.1 ppm)	[127]
2	Zinc phosphide nanowires	*Escherichia coli*	Greater than 4 log reduction	[128]
3	Silver nanomaterial in polysulfone membranes	*Escherichia coli*	90 percent efficiency (silver leaching 2 μg L^–1^)	[129]
4	Silver nanomaterial loaded chitosan cryogels	*Bacillus* *subtilis* and *Escherichia coli*	3 log reduction (silver content –7.5 mg/g)	[130]
5	Carbon powder-Vanadium Tetrasulfide nanocomposite	*Escherichia coli*	9.7 log reduction (at 0.1 g/L)	[131]
6	Titanium dioxide-Iron oxide nanocomposite	*Escherichia coli*	99.28% removal efficiency (initial concentration of bacteria: 10 mg/mL)	[132]
7	Carbon nanoparticles	*Escherichia coli bacteria*	6 log reduction (25 mg/50 mL concentration)	[133]

**Table 4:** The toxicity as well as the function of different commonly used nanomaterials in the aqueous environment.

Sl. No.	Nanomaterials	Concentration/Toxicity	Remediation Measure	Reference
1	Nano-zerovalent iron	Six bacteria were examined for nZVI concentration, and the EC_50_ of the bacteria against pristine pyrophoric nano zerovalent iron was noted to be 0.30–1.10 g/L. Irrespective of the difference in EC_50_ values, the development of malondialdehyde displayed a similar tendency for all the tested bacteria.	The iron present in the ageing nano zerovalent iron underwent a reduction to the Fe^2+^ ion, which could decrease the pollutants.	[154,155]
2	Graphene oxide	With graphene oxide only, lethal effects on *Artemia salina* have been noted just at a high concentration of graphene oxide (500 ppm), however sublethal toxicity (hindrance of growth) has been noted while the graphene oxide loading was as little as 1 ppm, which might be brought about by the oxidative stress stimulated by graphene oxide. In the acute toxicity analysis on *Artemia salina,* the pure graphene showed no toxicity at 10 mg/L maximal concentration. Other nanomaterials in other crustacean species confirmed a similar toxicity tendency.	Adsorbing heavy metal ions as well as organic pollutants.	[156,157]
3	Zinc oxide nanoparticles	The median lethal concentrations of 20 nm zinc oxide nanoparticles towards *Hydra magnipapillata* have been 7.0, 8.70, and 55.30 μg/mL after exposing to 96, 72, and 48 h, respectively, when the median lethal concentrations of 100 nm zinc oxide nanoparticles have been 9.90, 14.90, and 262.0 μg, respectively.	Absorbing different elements like Cd, Ni, Cu, Pb, Hg, Mo, Al, and As, altering their speciation in the media and thus the bioavailability.	[158,159]
4	Titanium dioxide nanoparticles	When the titanium dioxide nanoparticle concentration attained 10 ppm, the speediness of sperm cells reduced, also superoxide dismutase activity as well as the total glutathione level got enhanced.	Resilient absorbability to As(V), As(III), and Cd.	[160,161]
5	Silver nanoparticles	When the silver nanoparticle concentration was 0.1 ppm, the biological behavior of *Corbicula fluminea* has been restrained. When the silver nanoparticle concentration attained 2 ppm, the physiological metabolism of *Corbicula fluminea* has been restrained again.	Silver nanoparticles have the ability for absorbing the heavy metal ions in water, like zinc and copper ions.	[162,163]
6	Silver nanoparticle	Fresh water fish *Labeo rohita* exposed to the nanoparticle (25 mg/L) for 21 days. Survival of fish was unaltered. Exhibited less toxicity.	Activated carbon can be used as an adsorbent to remove silver from water.	[164,165]
7	Nickel nanoparticle	Fresh water fish *Labeo rohita* exposed to the nanoparticle (25 mg/L) for 21 days. Survival of fish was unaltered. Remarkable reduction in growth and hemoglobin observed. Significant total protein rise noted.	Nanocomposite of magnetic hydroxyapatite can be used as an adsorbent for the removal of copper nickel (Ni(II)) from water.	[164,166]
8	Cobalt oxide Nanoparticle	Fresh water fish *Labeo rohita* exposed to the nanoparticle (25 mg/L) for 21 days. Survival of fish was unaltered. Significant total protein rise noted.	Zinc oxide nanopowder can be used for the cobalt oxide nanoparticle removal.	[164,167,168]
9	Chromium oxide nanoparticle	Fresh water fish *Labeo rohita* exposed to the nanoparticle (25 mg/L) for 21 days. Cr_3_O_4_ nanoparticles caused early fish mortalities. Remarkable reduction in growth and hemoglobin. Significant total protein rise noted.	Superparamagnetic iron oxide nanoparticles can be used for chromium oxide removal.	[164,169]
10	Silver nanoparticles	*Prochilodus lineatus* fish exposed to 2.5 and 25.0 µg L^−1^ nanoparticle for 5 and 15 days. ACAP reduced in liver, all antioxidant enzymes activities increased, muscle protein concentration reduced, and glycogen content enhanced in liver and muscle.	Activated carbon can be used as an adsorbent to remove silver from water.	[165,170]
